# The Association between Symptoms of Depression and Anxiety, Quality of Life, and Diabetic Kidney Disease among Chinese Adults: A Cross-Sectional Study

**DOI:** 10.3390/ijerph20010475

**Published:** 2022-12-28

**Authors:** Yan Shen, Yi Chen, Shichun Huang, Xuejie Yao, Yashpal S. Kanwar, Ming Zhan

**Affiliations:** 1 School of Medicine, Ningbo University, Ningbo 315000, China; 2 Department of Medicine, Ningbo First Hospital, Zhejiang University, Ningbo 315000, China; 3 Department of Pathology, Northwestern University, Chicago, IL 60611, USA; 4 Department of Medicine, Northwestern University, Chicago, IL 60611, USA; 5 China Health Institute, University of Nottingham Ningbo China, Ningbo 315100, China

**Keywords:** diabetic kidney disease, depression, anxiety, quality of life

## Abstract

Depression and anxiety are common comorbid symptoms among patients with diabetic kidney disease (DKD). Little is known about the influence of poor psychological conditions on the disease progression and quality of life (QOL) in DKD patients. This study aimed to investigate the prevalence of, and risk factors for, depression and anxiety in Chinese DKD patients, and to analyze their impact on the renal function, proteinuria, and QOL. A total of 620 adult patients with Type 2 diabetes and DKD being treated at a tertiary hospital in East China were recruited. Depression and anxiety symptoms were assessed by the Zung Self-Rating Depression Scale and Anxiety Scale. Among the DKD participants, 41.3% had symptoms of depression and 45.0% had anxiety symptoms. A poor education, physical inactivity, stroke, low serum albumin, CKD stage 3–4, macroalbuminuria, and a poor QOL were independent risk factors for depression in the DKD patients. Whereas a higher education, physical inactivity, diabetic retinopathy and neuropathy, low hemoglobin, CKD stage 3–4, and a poor QOL were risk factors for anxiety. Depression and anxiety scores among the DKD patients were negatively correlated with the eGFR and QOL scores. Moreover, depression and anxiety symptoms were independent risk factors for DKD patients with CKD stage 3–4 and a poor QOL. Our findings suggest a high prevalence of depression and anxiety among Chinese DKD patients, and the severity of psychological symptoms is closely linked to the deterioration of renal function and the QOL. The early screening and intervention of psychopathological disorders is thus strongly recommended for improving the QOL and clinical outcomes among DKD patients.

## 1. Introduction

Diabetic kidney disease (DKD) is a common reno-microvascular complication of diabetes mellitus, and 30 to 40% of people with diabetes progress to DKD [1]. According to the report released by the International Diabetes Federation, the number of patients with diabetes worldwide were 436 million in 2019, and this is estimated to reach 700 million by 2045 [2]. Moreover, China has currently the largest number of diabetic patients, accounting for approximately 116 million among the general population [2]. With the continuous increase in the incidence of diabetes, the prevalence of DKD is expected to rise. Interestingly, it seems that the incidence of DKD in China has surpassed chronic glomerulonephritis, and thus it has become the major contributing factor for chronic kidney disease (CKD) since 2011 [3].

Depression and anxiety are common psychological disorders in patients with chronic illnesses. Recent studies have shown that approximately one-third of patients with diabetes suffers from depression disorders [4]. Patients with diabetes are twice as likely to develop depression and anxiety, compared to the general population [5]. Of equal importance is that diabetic complications including DKD are closely associated with depression [4]. Cohort studies have also reported that depression in patients with DKD is at an increased risk of progressing at a faster rate to end-stage renal disease (ESRD) [6]. Similarly, patients with CKD are often seen to have more depressive and anxiety symptoms, which often accompany adverse clinical outcomes, including the accelerated loss of kidney function, frequent hospitalizations, and high mortality rates, as well as poor quality of life [7,8]. Previous studies which focused on patients with diabetes or CKD identified several socio–demographic and clinical factors that could predispose these patients to developing psychopathological distress [4,9]. However, information about the prevalence and risk factors for depression and anxiety among DKD patients, and the association of DKD with a patient’s quality of life (QOL) remain poorly understood, especially in China. Also, little is known about whether and how psychopathological stress influences the progression of DKD.

Considering these findings, it seems essential to comprehensively investigate the QOL and psychological status of patients with DKD, to implement ameliorating supportive strategies for the prevention of depression and anxiety in these patients. 

The aim of this study was to assess the prevalence and the relevant influencing risk factors for depression and anxiety among Chinese patients with DKD, and to analyze the predictive value of risk factors. We also explored the relationship between psychopathological issues and clinical outcomes that were adversely affected. The clinical outcomes included renal function, albuminuria, and the QOL in DKD patients. With these results, evidence was provided to offer suggestions for suitable and more optimal strategies for the management of DKD patients.

## 2. Methods

### 2.1. Research Design and Participants

This cross-sectional study was conducted during the period from January 2021 to August 2021 at the comprehensive tertiary hospital, Ningbo First Hospital. This hospital has four branches located in four different districts of Ningbo City in East China. The participants consisted of a total of 646 patients who were diagnosed with Type 2 diabetes mellitus (T2DM) and DKD. They were all regularly treated in the department of medicine from the four branches of the Ningbo First Hospital, with data from 620 participants included for analysis in this study. Those with incomplete or ineligible data were not included in the study. The patients with T2DM were chosen by following the World Health Organization’s diagnostic criteria for diabetes [10]. The diagnosis of DKD was based on the guidelines provided in the National Kidney Foundation–Kidney Disease Outcome Quality Initiative (NKF–K/DOQI) [11]. The patients with CKD, which was caused by diabetes, presented with major manifestations regarding the urinary albumin/creatinine ratio (UACR) ≥30 mg/g, and/or the estimated glomerular filtration rate (eGFR) of <60 mL min^−1^ (1.73 m^2^)^−1^ symptoms for more than 3 months. The inclusion criteria that were considered were that participants had to be at least 18 years of age with a diagnosis of type 2 diabetes and DKD. Exclusion criteria included type 1 diabetes, past medical history of mental illness or cognitive dysfunction, patients on dialysis, and other types of primary or secondary renal diseases. The study was approved by the Ethics Committee of Ningbo First Hospital (Ethics Approval No: 2021-R019), and was performed in accordance with the Declaration of Helsinki treaty. All participants provided written informed consent prior to the enrollment in the study.

### 2.2. Data Collection

Socio–demographic and clinical data of the participants were collected during their visit for psychological assessment. The renal function was evaluated for the estimated glomerular filtration rate (eGFR), which was calculated using the Chronic Kidney Disease Epidemiology Collaboration (CKD-EPI) equation [12]. DKD was categorized into five CKD stages based on the eGFR. According to the Kidney Disease Improving Global Outcomes (KDIGO) guidelines [13], CKD stages 1–2 were defined as an eGFR of ≥60 mL/min/1.73 m^2^ and CKD stages 3–4 as an eGFR of 15–59 mL/min/1.73 m^2^. The stages of albuminuria based on the UACR were determined as follows: normoalbuminuria, UACR < 30 mg/g Cr; microalbuminuria, 30 ≤ UACR < 300 mg/g Cr; and macroalbuminuria, UACR ≥ 300 mg/g Cr. The criteria for physically active patients were that participants were considered physically active if they were able to walk for at least 30 min per day [14]. Body mass index (BMI) was computed as the ratio of body weight to the square of height (kg/m^2^).

### 2.3. The Assessment of Depression and Anxiety Symptoms

Depression and anxiety symptoms were assessed using self-questionnaires, according to the Zung Self-Rating Depression Scale [15] (SDS) and the Self-Rating Anxiety Scale (SAS) [16], respectively. The Chinese version of these two scales has a high validity and reliability and has been widely used in the Chinese population, including for patients with diabetes or kidney diseases [17,18]. Both questionnaires consisted of 20 items which were scored on a scale of 1 to 4. The original score for the 20 questions was multiplied by 1.25, and the integer portion was retained as a standard score. Higher scores indicated higher levels of anxiety or depression. The cut-off value of the SDS standard score was 53 points, and 53–62 points was defined as mild depression, 63–72 points was defined as moderate depression, and ≥73 points was defined as severe depression. The cut-off value of the SAS standard score was 50 points, of which a score of 50–59 was defined as mild anxiety, a score of 60–69 was defined as moderate anxiety, and a score of ≥70 was defined as severe anxiety [15]. 

### 2.4. The Assessment of Quality of Life

The health-related quality of life (QOL) was measured using the EuroQoL five-dimensional three-level (EQ-5D–3L) instrument, which contains a health state description. The health state description covers three levels of the five dimensions, resulting in 243 possible health states. Each health state can be transformed into a health utility score (i.e., EQ-5D–3L index score) that ranges from −0.149 to 1.0; the Chinese version of the EQ-5D–3L value sets were used in this study [19]. In general, the higher the EQ-5D–3L index score, the better is the individual’s QOL. Based on the EQ-5D–3L index score, we defined the normal quality of life as an EQ-5D–3L index of ≥0.665, and a low or poor quality of life as an EQ-5D–3L index of <0.665; this formulation has been applied in a previous study of the Chinese population [20]. 

### 2.5. Statistical Analysis

All the data were analyzed with the STATA 16.0 software. The continuous variables were expressed as the mean ± SD, while categorical variables were described as percentages, as appropriate. The groups were compared using an independent t-test, a Mann–Whitney U test and a chi-square test analysis, as considered appropriate. A multiple logistic regression was employed to identify independent risk factors that are associated with depression and anxiety among DKD patients, and a receiver operating characteristic (ROC) curve was used to evaluate the prediction effect of these risk factors on the psychological status of the DKD patients. To further investigate whether poor psychological conditions affected the eGFR, UACR, and QOL in patients with DKD, we performed a Kendall Rank correlation analysis using the eGFR, UACR, and EQ-5D–3L index scores as the dependent continuous variables, as well as a multiple logistic regression analysis defining the eGFR as ≥60 mL/min/1.73 m^2^ or 15–59 mL/min/1.73 m^2^, UACR as ≥300 mg/g or <300 mg/g, and the EQ-5D–3L index score as ≥0.655 or <0.665 as dependent binary variables, respectively. All tests were two-tailed with the significance considered as *p* < 0.05.

## 3. Results

### 3.1. General Characteristics of Participants

Socio–demographic and clinical characteristics of the study participants are included in Table 1. Among 620 participants with T2DM and DKD, 323 (52.1%) were male and 297 (47.9%) were female. Most of the patients were aged 50 and older (79.0%) and48.7% of the participants had poor education and were defined as having primary education or less, while 10.2% of the patients had high education level, and they had attained college education or above. Current smokers and alcohol drinkers comprised 26.8% and 17.1% of the participants, respectively. The prevalence of other diabetic complications and comorbidities among DKD patients was as follows: 81.5% were diagnosed with hypertension, 46.6% developed diabetic retinopathy, 71.1% had diabetic neuropathy, 33.7% had cardiac disease, and 10.5% had had a stroke. The distributions of DKD patients by the CKD staging parameters were 61.0 % for stages 1–2 and 39.0 % for stages 3–4. The mean serum albumin level was 37.16 g/L (37.16 ± 4.82 g/L), the mean levels of the eGFR and UACR were 70.22 mL/min/1.73 m^2^ (70.22 ± 28.56 mL/min/1.73 m^2^) and 344.81 mg/g (344.81 ± 368.12 mg/g), respectively. The QOL score showed that 24.8% of the patients with EQ-5D–3L had an index score < 0.655. 

### 3.2. The Prevalence of Symptoms of Depression and Anxiety

Among the total of 620 DKD patients, 256 (41.3%) had symptoms of depression, whereas 279 (45.0%) had anxiety symptoms, and 196 (31.6%) had both symptoms of depression and anxiety. Moderate to severe depression and anxiety status were noted in 131 (21.1%) and 128 (20.6%) of the patients, respectively, as shown in Table 2. These results indicated that almost half of the participants with DKD have a certain degree of depression or anxiety, suggesting a wide prevalence of impaired psychological well-being in the DKD patients. Therefore, adding a psychological scale for evaluation may facilitate the early detection of comorbid psychopathological symptoms in patients with DKD [21]. 

### 3.3. Risk Factors for Depression and Anxiety in Patients with DKD

Univariate logistic regression analyses examining the risk factors related to depression and anxiety among participants are presented in Table 3. Compared with the non-depressed patients, those with symptoms of depression had certain characteristics including an older age, a lack of physical activity, a low education level, and diabetic comorbidities and complications, with more advanced CKD Stages (3–4 vs. 1–2), macroalbuminuria (UACR ≥ 300 mg/g), and they also happened to have a poor QOL (EQ-5D–3L < 0.665). Depressed patients also had lower levels of hemoglobin and serum albumin, and had higher BUN levels, compared with those without depression. Similar characteristics were found in the DKD patients who were comorbid with anxiety. The participants who were older and showed a lack of physical activity, had diabetic comorbidities and complications with CKD Stages 3–4, macroalbuminuria (UACR ≥ 300 mg/g), and a poor QOL (EQ-5D–3L < 0.665) and they were more likely to show anxiety symptoms (*p* < 0.05). In addition, higher uric acid and BUN levels and a lower hemoglobin and serum albumin were also significantly associated with anxiety (*p* < 0.05). 

Variables that were statistically significant in the univariate analyses were selected and further analyzed by multivariable logistic regression, to adjust the potential confounding factors (Table 4). We observed that low education levels, physical inactivity, stroke, low serum albumin levels, CKD Stages 3–4, macroalbuminuria, and a poor QOL were independently associated with depression among DKD patients. Compared with the CKD stages 1–2 patients, we noted an increase of 1 in the odds ratio of depression occurring in patients with CKD stages 3–4 (OR = 2.051, 95% CI = 1.291–3.258, *p* = 0.002). The risk of depression in patients with low quality of life scores was increased five times compared to patients with a normal quality of life (OR = 6.003, 95% CI = 3.473–10.377, *p* < 0.001). On the other hand, high education levels, physical inactivity, neuropathy, retinopathy, low hemoglobin levels, CKD stages 3–4, and a poor QOL were independently associated with anxiety. The risk of anxiety in CKD stages 3–4 patients was 1.3 times higher in comparison to CKD stages 1–2 patients (OR = 2.282, 95% CI = 1.393–3.738, *p* < 0.001). Similarly, participants with low quality of life scores were seven times more likely to present with anxiety (OR = 7.062, 95% CI = 3.976–12.544, *p* < 0.001). 

### 3.4. The ROC Analysis

**Figure 1:**
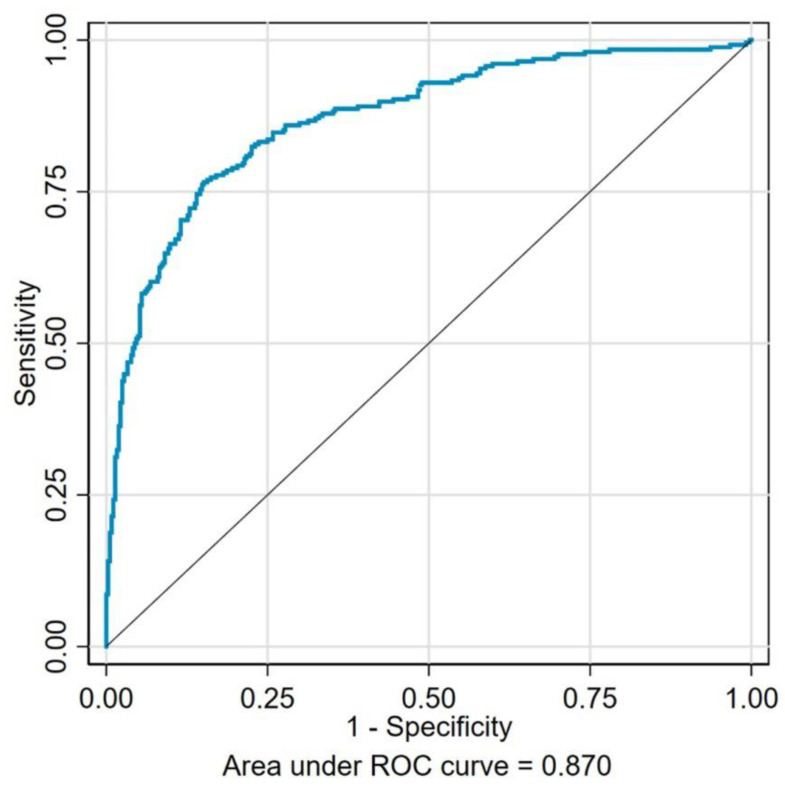
The receiver operating characteristics for seven risk factors (low education levels, physical inactivity, stroke, low serum albumin levels, CKD Stages 3–4, macroalbuminuria, and a poor QOL) combined to predict depression in DKD patients. The area under the curve was 0.870.

**Figure 2:**
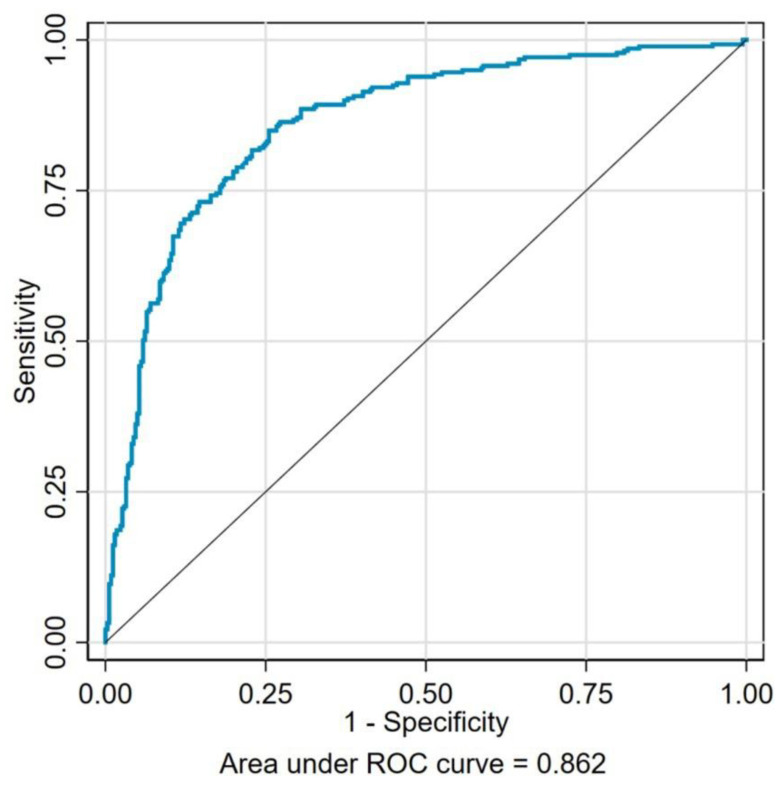
The receiver operating characteristics for seven risk factors (high education level, physical inactivity, neuropathy, retinopathy, low hemoglobin concentration, CKD stages 3–4, and a poor QOL) combined to predict anxiety in the DKD patients. The area under the curve was 0.862.

Based on the results of the multivariate logistic regression analysis, the receiver operating characteristic (ROC) curve was plotted to analyze the predictive value of the independent risk factors for depression and anxiety. Figure 1 represents the area under ROC curve (AUC) as 0.870, which includes a combination of seven identified risks factors of depression (low education levels, physical inactivity, stroke, low serum albumin levels, CKD Stages 3–4, macroalbuminuria, and a poor QOL). As shown in Figure 2, the AUC of those seven risk factors (high education levels, physical inactivity, neuropathy, retinopathy, low hemoglobin levels, CKD stages 3–4, and a poor QOL) combined for predicting anxiety was 0.862. These results indicated a high and reliable predictive value of these identified risk factors for depression and anxiety among the DKD patients.

### 3.5. The Association of Poor Psychological Status with Kidney Function, Albuminuria, and Quality of Life among DKD Patients

As shown in Table 5, the Kendall Rank correlation analysis revealed that the eGFR (r = −0.588, *p* < 0.001) and EQ-5D–3L index scores (r = −0.690, *p* < 0.001) were negatively associated with the Self-Rating Depression Scale (SDS) score. While the UACR was positively associated with the SDS score (r = 0.440, *p* < 0.001) when these were treated as continuous variables. Similarly, the eGFR (r = −0.535, *p* < 0.001) and EQ-5D–3L index scores (r = −0.646, *p* < 0.001) were also inversely correlated with the Self-Rating Anxiety Scale (SAS) score, and the UACR was positively associated with the SAS score (r = 0.388, *p* < 0.001). 

Furthermore, in a multivariable logistic regression analysis (Table 6), depression and anxiety were confirmed to be independent risk factors for deteriorating kidney function (eGFR 15–59 mL/min/1.73 m^2^), after adjustment for factors, such as, sex, age, education, and diabetic complications that may possibly impact kidney function. As shown in Table 6, participants with depressive symptoms had twice the risk of deteriorating kidney function (OR = 2.00, 95% CI = 1.20–3.34, *p* = 0.008), and DKD patients with anxiety symptoms also had nearly twice the risk of deteriorating kidney function (OR = 1.82, 95% CI = 1.09–3.02 *p* = 0.021). However, when all of the variables were entered into the model, depression or anxiety did not have an independent association with macroalbuminuria (UACR > 300 mg/g). In the multivariate model, depression and anxiety were also found to be predictors of poor quality of life (EQ-5D–3L index score < 0.065). Participants with depressive symptoms were 4.96 times more likely to have poor quality of life, compared with those without depression (OR = 4.96, 95% CI = 2.74–8.99, *p* < 0.001). Those with anxiety symptoms were 5.90 times the odds of having poor quality of life (OR = 5.90, 95% CI = 3.25–10.71, *p* < 0.001). 

All these results demonstrated that the depression and anxiety scores of the DKD patients were negatively correlated with the eGFR and quality of life scores. Depression and anxiety symptoms were independent risk factors for patients with CKD stage 3–4 and a poor QOL. These findings suggested that the severity of the patients’ depression and anxiety is closely linked to the deterioration of their renal function and the QOL.

## 4. Discussion

This study discovered that the prevalence rates of symptoms are 41.3% for depression and 45% for anxiety, and this indicates a high prevalence of depression and anxiety states in Chinese DKD patients. These results are comparable to those reported in a Japanese study where 37.3% of DKD patients had depressive symptoms [4], while data pertaining to the prevalence of anxiety in DKD patients are very limited. On the other hand, a series of studies have shown that 11.5–26.3% of diabetes patients have depressive symptoms, and 27.6–30.5% of diabetes patients presented with symptoms of anxiety [22,23,24]. Based on these data, symptoms of depression and anxiety may be more prevalent in diabetic patients with DKD, as compared with those without DKD [24,25]. Similarly, diabetic complications were also found to be associated with a further reduced QOL in diabetes patients [26], all of which indicate an additional negative impact of diabetic kidney complications on the psychopathological state and the QOL among diabetes patients. 

In our current model, low education levels, physical inactivity, stroke, lower serum albumin levels, CKD Stages 3–4, macroalbuminuria, and a poor QOL were identified as the risk factors that were significantly associated with depression in DKD patients, while factors notably associated with anxiety were high education level, physical inactivity, neuropathy, retinopathy, lower hemoglobin levels, CKD stages 3–4, and a poor QOL. To our knowledge, this is the first study identifying the independent risk factors associated with depression and anxiety symptoms in Chinese patients with DKD. To further confirm the prediction efficiency of the combination of risk factors, we drew the ROC curve, and both of the AUC were greater than 0.7, which indicated that the above-identified risk factors have a relatively good predictive value for the occurrence of psychopathological disorders in DKD patients. Moreover, our study demonstrated that depression and anxiety were both associated with deteriorating kidney function and a poor QOL, indicating that a poor psychopathological state may be an important factor that exacerbates somatic symptoms and the deterioration of kidney function in DKD patients as well as impairing the health-related quality of life. 

Several studies have been performed to explore the risk factors for anxiety and depression in patients with diabetes or CKD. Consistent with previous findings [9,27], we observed that patients with a lower educational level were more likely to develop depressive symptoms; whereas a higher education level increases the risk of having anxiety symptoms. Less-educated individuals may lack the accessibility to reliable information about diabetes and its complications, and this may cause them to worry about their disease status, thereby increasing the risk of developing depression [9]. Pu et al. speculated that patients with a higher education may be more capable of perceiving and recognizing the risk of DKD and they might at times be misled by misinformation, which may cause them to be vulnerable to anxiety [28]. Therefore, for patients with chronic diseases, including DKD, it is important that physicians provide more comprehensive and educational information in order to improve the awareness of the relevant disease and also the potential psychological repercussions. Generally, it is well recognized that lifestyle modification is strongly related to less psychological distress [25]. Our findings complement other studies showing that the lack of physical activity is closely associated with an increased risk of both depression and anxiety among patients with DKD. Cumulative evidence suggests that immune system malfunctions and chronic inflammation are linked to the development of depression [25,29]. In this regard, the protective effect of regular physical exercise on mental disorders in patients with chronic diseases may be attributed to the potential anti-inflammatory and immune function-boosting effects [29].

Among the clinical factors we studied, diabetic retinopathy was associated with a higher risk of anxiety, this might be because vision impairment has a detrimental impact on daily activities, social life, and quality of life [30]. Rajput et al. also observed that nephropathy, retinopathy, and ischemic heart disease were significantly associated with depression and anxiety [24]. In addition, we observed that a comorbid stroke influenced mental well-being and increased depression levels in DKD patients. Similarly, a recent study from China has demonstrated that cerebrovascular disease in CKD populations was associated with the increased incidence of depressive symptoms [28]. This correlation may be related to inflammation, autonomic nervous system dysfunction, and platelet aggregation enhancement, all of which have been proposed to explain the link between depression and strokes [31,32]. In this study, a low serum albumin level was noted to be a predictive factor for depression among participants. A low level of serum albumin may reflect malnutrition, which has previously also been shown to be associated with depression and poor clinical outcomes in dialysis patients [33]. Similarly, the present study revealed that low hemoglobin levels were independently correlated with a high anxiety rate, which concurs with several previous studies [34,35]. A low serum hemoglobin, often classified as anemia, which is an important complication of CKD that increases fatigue and dyspnea and negatively affects social activity, may thus be linked to psychological disorders [28,35].

DKD is typically characterized by an increased urinary albumin excretion and the progressive deterioration in kidney function, which ultimately may result in ESRD [1]. In the present study, comparing the DKD patients with CKD Stages 1–2 to those with CKD Stages 3–4, we found that the latter group had a significantly higher risk of depression or anxiety. Similarly, a cross-sectional study of 2212 diabetes patients in Japan demonstrated that the later stages of DKD were incrementally associated with more a severe and higher risk of depression [4]. In another study by Campbell et al., participants with a lower eGFR category (eGFR ≤ 29 mL/min/1.73 m^2^) had twice the risk of depression as compared to those with a high eGFR (≥90 mL/min/1.73 m^2^) among patients with diabetes and CKD [36]. On the other hand, we also noted that macroalbuminuria was a risk factor that was associated with depression in DKD patients; these results are consistent with other studies showing that there is a significant correlation between albuminuria and the severity of depressive symptoms in patients with CKD and DKD [4,27]. On the other hand, in the regression model, we observed that patients with DKD who had either depression or anxiety were more likely to have advanced stages of CKD and a poor QOL. Moreover, among the DKD patients with psychological symptoms in this study, a high proportion of the patients suffered from both depression and anxiety. It is conceivable that the DKD patients with a combination of depression and anxiety have a higher risk of kidney function deterioration and a poorer QOL, as compared with those who have only one or no psychological disorders; this speculation needs to be confirmed by further studies. Also, although this cross-sectional study was unable to clarify the causal relationship of the eGFR and albuminuria with depression and anxiety, several cohort studies have indicated that depressive symptoms may accelerate the decline of kidney function and contribute to adverse renal disease outcomes [6,37,38]. A 3-year cohort study suggested that CKD patients with depressive symptoms had higher risk for progression to dialysis and an all-cause mortality [37]. Horiba Y et al. reported that the presence of depression has a potential impact on the progression to ESRD in patients with advanced DKD [6]. Zhang Z et al. also reported that depressive symptoms in Chinese adults were significantly associated with a higher risk of the rapid decline of kidney function [38]. The potential mechanisms by which psychological disorders contribute to the deterioration of kidney function were also addressed. Interestingly, several studies have indicated that depression and anxiety may enhance the activity of the hypothalamic–pituitary–adrenal (HPA) axis, which would further increase the glucocorticoid corticosterone levels and leading to the impairment of immune system [39,40]. On the other hand, depressive symptoms are also associated with higher inflammatory cytokines levels, such as C-reactive protein and interleukin-6, which may contribute to systemic inflammation and deteriorating kidney function [37,38]. However, the underlying mechanisms controlling the contribution of depression and anxiety to the loss of kidney function remain largely unknown, and this is certainly worthy of further investigation.

Health-related quality of life has increasingly become an important outcome in chronic diseases, including DKD. In this regard, the validated EQ-5D–3L instrument has been shown as a reliable tool to evaluate the QOL of patients with diabetes and its associated complications [41]. Our findings indicated that a poor QOL can increase the risk of depression and anxiety symptoms in patients with DKD. A poor QOL may have a negative impact on an individual’s independence in daily life, the ability to work and various aspects of health, and this may further be associated with developing psychiatric disorders [8]. A recent study using EQ-5D–3L to evaluate the QOL in diabetic patients showed that the QOL was worse in patients with additional diabetic complications [26], and improving the QOL may promote mental health in patients with DKD [25]. Conversely, our study also suggested that depression and anxiety were independently related to a poor QOL. The potential causes of depression and anxiety that adversely affect the QOL in DKD patients are likely to be linked to medication non-compliance, poor nutrition, and negative clinical outcomes [27,42]. These findings highlight the significant association between psychological disorders and the QOL in DKD patients. Comprehensive strategies, including early assessment and proactive treatment of depression and anxiety along with the use of emerging medications, may substantially improve the QOL and clinical outcomes in DKD patients (Table 7).

## 5. Conclusions

This study indicates that depression and anxiety symptoms have a high prevalence in patients with DKD in China. Poor education, physical inactivity, stroke, low concentration of serum albumin, CKD stage 3–4, macroalbuminuria, and a poor QOL were independent risk factors for depression in DKD patients. The factors independently associated with anxiety were high education level, physical inactivity, diabetic retinopathy and neuropathy, low hemoglobin level, CKD stage 3–4, and a poor QOL. In addition, results of our study implied that the severity of the patients’ depression and anxiety are closely linked to the deterioration of their renal function and the QOL. Therefore, it is essential to improve the awareness of psychological comorbidities among DKD patients in China. The findings of our study suggest the importance of endocrinologists, nephrologists, and psychologists working together to initially screen and evaluate DKD patients for psychological disturbances, and take supportive measures to maintain good mental health. The comprehensive strategies for the management of both somatic and psychological disorders may effectively delay the disease progression, improve the QOL, and prevent adverse clinical outcomes in DKD patients.

## Figures and Tables

**Table 1:** Socio–demographic and clinical characteristics of the participants (n = 620).

Characteristics	Total (n = 620)
Age group, years, n (%)	
≤50	130 (21.0)
50–60	161 (26.0)
60–70	170 (27.4)
>70	159 (25.7)
Gender, n (%)	
Male	323 (52.1)
Female	297 (47.9)
Education level, n (%)	
Low, primary school and below	302 (48.7)
Medium, junior and high school	255 (41.1)
High, college and above	63 (10.2)
BMI level, kg/m^2^, n (%)	
<24	300 (48.4)
24–27.9 (Overweight)	239 (38.6)
≥28 (Obese)	81 (13.1)
Smoking, n (%)	166 (26.8)
Alcohol use, n (%)	106 (17.1)
Physical activity, n (%)	224 (36.1)
Comorbidities and complications, n (%)	
Hypertension	505 (81.5)
Retinopathy	289 (46.6)
Neuropathy	441 (71.1)
Cardiac disease	209 (33.7)
Stroke	65 (10.5)
eGFR, mL/min/1.73 m^2^	70.22 ± 28.56
CKD stage, n (%)	
CKD1–2 (≥60 mL/min/1.73 m^2^)	378 (61.0)
CKD3–4 (15–59 mL/min/1.73 m^2^)	242 (39.0)
UACR, mg/g	344.81 ± 368.12
UACR stage, n (%)	
30 ≤ UACR < 300 mg/g	374 (60.3)
UACR ≥ 300 mg/g	246 (39.7)
HbA1c,%	8.15 ± 2.04
Hemoglobin, g/L	120.18 ± 45.79
Serum albumin, g/L	37.16 ± 4.82
Uric Acid, mmol/L	367.18 ± 110.83
BUN, mmol/L	8.30 ± 14.73
TG, mmol/L	1.95 ± 3.43
TC, mmol/L	4.30 ± 1.41
HDL, mmol/L	1.11 ± 0.32
LDL, mmol/L	2.99 ± 1.05
EQ-5D–3L index score	
EQ-5D–3L ≥ 0.655	466 (75.2)
EQ-5D–3L < 0.655	154 (24.8)

Abbreviations: BMI, body mass index; eGFR, estimated glomerular filtration rate; CKD, chronic kidney disease; UACR, urine albumin to creatinine ratio; HbA1c, hemoglobin A1c; BUN, blood urea nitrogen; TG, Triglyceride; TC, Total cholesterol; HDL, high-density lipoprotein; LDL, low-density lipoprotein. EQ-5D–3L, EuroQol Five-Dimensional Three-Level Health Questionnaire. The continuous variables are shown as the mean ± SD and the categorical variables are shown as a number (percentage).

**Table 2:** The prevalence of symptoms of depression and anxiety among the DKD patients (n = 620).

	Average Score	Normal, n (%)	Depression or Anxiety Present, n (%)			
			Total	Mild	Moderare	Severe
Depression	48.6 ± 20.6	364 (58.7)	256 (41.3)	125 (20.2)	102 (16.5)	29 (4.7)
Anxiety	48.5 ± 12.1	341 (55.0)	279 (45.0)	151 (24.4)	101 (16.3)	27 (4.4)

**Table 3:** Socio–demographic and clinical factors associated with depression and anxiety in the DKD patients.

Characteristics	Depression			Anxiety		
	Present	Absent	*p* Value	Present	Absent	*p* Vaule
Age group, years, n (%)			<0.001			0.012
≤50	32 (12.5)	98 (26.9)		50 (17.9)	80 (24.5)	
50–60	57 (22.3)	104 (28.6)		67 (24.0)	94 (27.6)	
60–70	73 (28.5)	97 (26.6)		73 (26.2)	97 (28.4)	
>70	94 (36.7)	65 (17.9)		89 (31.9)	70 (20.5)	
Gender, n (%)			0.090			0.094
Male	123 (48.0)	200 (54.9)		135 (48.4)	188 (55.1)	
Female	133 (52.0)	164 (45.1)		144 (51.6)	153 (44.9)	
Education level, n (%)			<0.001			0.003
Low, primary school and below	163 (63.7)	139 (38.2)		153 (54.8)	149 (43.7)	
Medium, junior and high school	78 (30.5)	177 (48.6)		94 (33.7)	161 (47.2)	
High, college and above	15 (5.9)	48 (13.2)		32 (11.5)	31 (9.1)	
BMI level, kg/m^2^, n (%)			0.777			0.204
<24	123 (48)	177 (48.6)		145 (52.0)	155 (45.5)	
24–27.9 (overweight)	102 (39.8)	137 (37.6)		103 (36.9)	136 (39.9)	
≥28 (obese)	31 (12.1)	50 (13.7)		31 (11.1)	50 (14.7)	
Smoking, n (%)	67 (26.2)	99 (27.2)	0.776	71 (25.4)	95 (27.9)	0.500
Alcohol use, n (%)	42 (16.4)	64 (17.6)	0.702	45 (16.1)	61 (17.9)	0.563
Physical activity, n (%)	47 (18.4)	177 (48.6)	<0.001	47 (16.8)	177 (51.9)	<0.001
Comorbidities and complications, n (%)						
Hypertension	225 (87.9)	280 (76.9)	0.001	240 (86.0)	265 (77.7)	0.008
Retinopathy	140 (54.7)	149 (40.9)	0.001	164 (58.8)	125 (36.7)	<0.001
Neuropathy	200 (78.1)	241 (66.2)	0.001	223 (79.9)	218 (63.9)	<0.001
Cardiac disease	128 (50.0)	81 (22.3)	<0.001	125 (44.8)	84 (24.6)	<0.001
Stroke	47 (18.4)	18 (4.9)	<0.001	44 (15.8)	21 (6.2)	<0.001
eGFR, mL/min/1.73 m^2^	54.36 ± 24.22	81.37 ± 26.02	<0.001	58.1 ± 26.69	80.14 ± 26.14	<0.001
CKD stage			<0.001			<0.001
CKD1–2 (≥60 mL/min/1.73 m^2^)	93 (36.3)	285 (78.3)		112 (40.1)	266 (78)	
CKD3–4 (15–59 mL/min/1.73 m^2^)	163 (63.7)	79 (21.7)		167 (59.9)	75 (22)	
UACR, mg/g	477.25 ± 381.47	251.67 ± 328.31	<0.001	445.25 ± 381.20	262.64 ± 335.94	<0.001
UACR stage, n (%)						
UACR < 300 mg/g	100 (39.1)	274 (75.3)	<0.001	123 (44.1)	251 (73.6)	<0.001
UACR ≥ 300 mg/g	156 (60.9)	90 (24.7)		156 (55.9)	90 (26.4)	
HbA1c, %	8.34 ± 2.05	8.02 ± 2.03	0.021	8.40 ± 2.11	7.95 ± 1.97	0.003
Hemoglobin, g/L	111.19 ± 19.03	126.50 ± 56.78	<0.001	110.13 ± 19.58	128.40 ± 57.90	<0.001
Serum albumin, g/L	34.61 ± 4.68	38.95 ± 4.05	<0.001	35.52 ± 4.86	38.50 ± 4.35	<0.001
Uric Acid, mmol/L	375.46 ± 119.87	361.37 ± 103.78	0.180	380.17 ± 122.76	356.56 ± 98.94	0.024
BUN, mmol/L	8.91 ± 4.64	7.87 ± 18.82	<0.001	8.55 ± 4.38	8.10 ± 19.47	<0.001
TG, mmol/L	2.0 ± 2.43	1.92 ± 3.99	0.242	1.85 ± 1.24	2.04 ± 4.49	0.194
TC, mmol/L	4.30 ± 1.41	4.29 ± 1.42	0.648	4.30 ± 1.51	4.30 ± 1.34	0.204
HDL, mmol/L	1.11 ± 0.3	1.12 ± 0.33	0.709	1.01 ± 0.30	1.12 ± 0.33	0.619
LDL, mmol/L	2.99 ± 1.1	2.98 ± 1.02	0.895	3.02 ± 1.13	2.95 ± 0.98	0.602
EQ-5D–3L index score			<0.001			<0.001
EQ-5D–3L ≥ 0.655	128 (50.0)	338 (92.9)		145 (52.0)	321 (94.1)	
EQ-5D–3L < 0.655	128 (50.0)	26 (7.1)		134 (48.0)	20 (5.9)	

Abbreviations: BMI, body mass index; eGFR, estimated glomerular filtration rate; CKD, chronic kidney disease; UACR, urine albumin to creatinine ratio; HbA1c, hemoglobin A1c; BUN, blood urea nitrogen; TG, Triglyceride; TC, Total cholesterol; HDL, high-density lipoprotein; LDL, low-density lipoprotein. EQ-5D–3L, EuroQol Five-Dimensional Three-Level Health Questionnaire. The continuous variables are shown as mean ± SD, categorical variables are shown as number (percentage). The threshold of statistical significance was defined as *p* < 0.05.

**Table 4:** The multivariate logistic regression analysis showing risk factors associated with depression and anxiety in the DKD patients.

Variable	Depression		Anxiety	
	OR (95% CI)	*p* Value	OR (95% CI)	*p* Value
Education level, n (%)				
Low, primary school and below	1		1	
Medium, junior and high school	0.426 (0.265–0.684)	<0.001	0.734 (0.464–1.162)	0.188
High, college and above	0.291 (0.130–0.649)	0.003	2.496 (1.222–5.100)	0.012
Physical activity	0.593 (0.370–0.950)	0.030	0.382 (0.242–0.601)	<0.001
Neuropathy			1.910 (1.156–3.156)	0.012
Retinopathy			1.790 (1.167–2.744)	0.008
Stroke	2.409 (1.121–5.174)	0.024		
Hemoglobin			0.983 (0.972–0.994)	0.003
Serum albumin	0.875 (0.826–0.926)	<0.001		
CKD stage				
CKD1–2 (eGFR ≥ 60 mL/min/1.73 m^2^)	1		1	
CKD3–4 (eGFR15–59 mL/min/1.73 m^2^)	2.051 (1.291–3.258)	0.002	2.282 (1.393–3.738)	0.001
UACR stage				
UACR < 300 mg/g	1		1	
UACR ≥ 300 mg/g	1.718 (1.038–2.843)	0.035	1.268 (0.772–2.083)	0.348
EQ-5D–3L index score				
EQ-5D–3L ≥ 0.655	1		1	
EQ-5D–3L < 0.655	6.003 (3.473–10.377)	<0.001	7.062 (3.976–12.544)	<0.001

Abbreviations: eGFR, estimated glomerular filtration rate; CKD, chronic kidney disease; UACR, urine albumin to creatinine ratio; EQ-5D–3L, EuroQol Five-Dimensional Three-Level Health Questionnaire. The threshold of statistical significance was defined as *p* < 0.05.

**Table 5:** The correlation analysis between the clinical indicators and depression and anxiety in the DKD patients.

Variable	Depression		Anxiety	
	r	*p* Value	r	*p* Valve
eGFR	−0.588	<0.001	−0.535	<0.001
UACR	0.440	<0.001	0.388	<0.001
EQ-5D–3L index score	−0.690	<0.001	−0.646	<0.001

Abbreviations: eGFR, estimated glomerular filtration rate; UACR, urine albumin to creatinine ratio; EQ-5D–3L, EuroQol Five-Dimensional Three-Level Health Questionnaire. The threshold of statistical significance was defined as *p* < 0.05.

**Table 6:** Multivariate logistic regression analysis of the association of poor psychological status with deteriorating kidney function, macroalbuminuria and poor quality of life in DKD patients.

Variable	eGFR15–59 mL/min/1.73 m^2^		UACR ≥ 300 mg/g		EQ-5D–3L < 0.665	
	OR (95% CI)	*p* Value	OR (95% CI)	*p* Value	OR (95% CI)	*p* Value
depression						
no	1		1		1	
yes	2.00 (1.20–3.34)	0.008	1.56 (0.94–2.57)	0.083	4.96 (2.74–8.99)	<0.001
anxiety						
no	1		1		1	
yes	1.82 (1.09–3.02)	0.021	1.18 (0.72–1.93)	0.522	5.90 (3.25–10.71)	<0.001

Abbreviations: eGFR, estimated glomerular filtration rate; UACR, urine albumin to creatinine ratio; EQ-5D– 3L, EuroQol Five-Dimensional Three-Level Health Questionnaire. The threshold of statistical significance was defined as *p* < 0.05.

**Table 7:** Comprehensive strategies for the management of patients with type 2 diabetes and DKD.

Multidisciplinary Collaboration	Lifestyle Modification	Diabetes Management	DKD Management	Psychological Management
Endocrinologist Nephrologist Psychologist Nutritionist	Diet control Alcohol and smoking cessation Weight control Physical exercises	Personalized blood glucose control Blood pressure control Cholesterol control Uric acid control	UACR and eGFR assessment ACE inhibitor or ARB Consider SGLT2i or GLP1-RA or Finerenone for kidney protection	Anxiety and depression assessment Quality of life assessment Psychological counselling and intervention

Abbreviations: UACR, urine albumin to creatinine ratio; eGFR, estimated glomerular filtration rate; DKD, diabetic kidney disease; ACE, angiotensin-converting enzyme; ARB, angiotensin II receptor blocker; SGLT2i, sodium-glucose cotransporter-2 inhibitor. GLP1-RA, glucagon-like peptide 1 receptor agonist.

## Data Availability

The data presented in this study are available on request from the corresponding author.

## References

[1] Umanath K., Lewis J.B. (2018). Update on Diabetic Nephropathy: Core Curriculum 2018. Am. J. Kidney Dis..

[2] Saeedi P., Petersohn I., Salpea P., Malanda B., Karuranga S., Unwin N., Colagiuri S., Guariguata L., Motala A.A., Ogurtsova K. (2019). Global and regional diabetes prevalence estimates for 2019 and projections for 2030 and 2045: Results from the International Diabetes Federation Diabetes Atlas, 9(th) edition. Diabetes Res. Clin. Pract..

[3] Zhang L., Long J., Jiang W., Shi Y., He X., Zhou Z., Li Y., Yeung R.O., Wang J., Matsushita K. (2016). Trends in Chronic Kidney Disease in China. N. Engl. J. Med..

[4] Takasaki K., Babazono T., Ishizawa K., Miura J., Uchigata Y. (2016). Relationship between diabetic nephropathy and depression: A cross-sectional analysis using the Diabetes Study from the Center of Tokyo Women’s Medical University (DIACET). BMJ Open Diabetes Res. Care.

[5] Khuwaja A.K., Lalani S., Dhanani R., Azam I.S., Rafique G., White F. (2010). Anxiety and depression among outpatients with type 2 diabetes: A multi-centre study of prevalence and associated factors. Diabetol. Metab. Syndr..

[6] Horiba Y., Ishizawa K., Takasaki K., Miura J., Babazono T. (2021). Effect of depression on progression to end-stage renal disease or pre-end-stage renal disease death in advanced diabetic nephropathy: A prospective cohort study of the Diabetes Study from the Center of Tokyo Women’s Medical University. J. Diabetes Investig..

[7] Tsai Y.-C., Chiu Y.-W., Hung C.-C., Hwang S.-J., Tsai J.-C., Wang S.-L., Lin M.-Y., Chen H.-C. (2012). Association of Symptoms of Depression with Progression of CKD. Am. J. Kidney Dis..

[8] Peng T., Hu Z., Guo L., Xia Q., Li D., Yang X. (2013). Relationship Between Psychiatric Disorders and Quality of Life in Nondialysis Patients with Chronic Kidney Disease. Am. J. Med. Sci..

[9] Sun N., Lou P., Shang Y., Zhang P., Wang J., Chang G., Shi C. (2016). Prevalence and determinants of depressive and anxiety symptoms in adults with type 2 diabetes in China: A cross-sectional study. BMJ Open.

[10] Grimaldi A., Heurtier A. (1999). Diagnostic criteria for type 2 diabetes. Rev. Prat..

[11] Kdoqi (2007). KDOQI Clinical Practice Guidelines and Clinical Practice Recommendations for Diabetes and Chronic Kidney Disease. Am. J. Kidney Dis..

[12] Levey A.S., Stevens L.A. (2010). Estimating GFR Using the CKD Epidemiology Collaboration (CKD-EPI) Creatinine Equation: More Accurate GFR Estimates, Lower CKD Prevalence Estimates, and Better Risk Predictions. Am. J. Kidney Dis..

[13] Sarnak M.J., Bloom R., Muntner P., Rahman M., Saland J.M., Wilson P.W., Fried L. (2015). KDOQI US Commentary on the 2013 KDIGO Clinical Practice Guideline for Lipid Management in CKD. Am. J. Kidney Dis..

[14] Al-Bekairy A., AbuRuz S., Alsabani B., AlShehri A., Al-Debasi T., Alkatheri A., Almodaimegh H. (2017). Exploring Factors Associated with Depression and Anxiety among Hospitalized Patients with Type 2 Diabetes Mellitus. Med. Princ. Pract..

[15] Song X., Chen L., Zhang T., Xiang Y., Yang X., Qiu X., Qiao Z., Yang Y., Pan H. (2021). Negative emotions, self-care activities on glycemic control in adults with type 2 diabetes: A cross-sectional study. Psychol. Health Med..

[16] Zung W.W. (1971). A Rating Instrument for Anxiety Disorders. Psychosomatics.

[17] Ran M.-S., Gao R., Lin J.-X., Zhang T.-M., Chan S.K.W., Deng X.-P., Zhang B.-Z., Zhang X.-F., Huang G.-P., Pu D.-S. (2022). The impacts of COVID-19 outbreak on mental health in general population in different areas in China. Psychol. Med..

[18] Hou Y., Li X., Yang L., Liu C., Wu H., Xu Y., Yang F., Du Y. (2014). Factors associated with depression and anxiety in patients with end-stage renal disease receiving maintenance hemodialysis. Int. Urol. Nephrol..

[19] Liu G.G., Wu H., Li M., Gao C., Luo N. (2014). Chinese time trade-off values for EQ-5D health states. Value Health.

[20] Han K., Yang S., Jia W., Wang S., Song Y., Cao W., Wang J., Liu M., He Y. (2020). Health-Related Quality of Life and Its Correlation with Depression Among Chinese Centenarians. Front. Public Health.

[21] Shah B.M., Gupchup G.V., Borrego M.E., Raisch D.W., Knapp K.K. (2008). Depressive symptoms in patients with type 2 diabetes in the ambulatory care setting: Opportunities to improve outcomes in the course of routine care. J. Am. Pharm. Assoc..

[22] Yu M.K., Weiss N.S., Ding X., Katon W.J., Zhou X.-H., Young B.A. (2014). Associations between Depressive Symptoms and Incident ESRD in a Diabetic Cohort. Clin. J. Am. Soc. Nephrol..

[23] Kaur G., Tee G.H., Ariaratnam S., Krishnapillai A., China K. (2013). Depression, anxiety and stress symptoms among diabetics in Malaysia: A cross sectional study in an urban primary care setting. BMC Fam. Pract..

[24] Rajput R., Gehlawat P., Gehlan D., Gupta R., Rajput M. (2016). Prevalence and predictors of depression and anxiety in patients of diabetes mellitus in a tertiary care center. Indian J. Endocrinol. Metab..

[25] Ninomiya H., Katakami N., Matsuoka T.-A., Takahara M., Nishizawa H., Maeda N., Otsuki M., Imagawa A., Iso H., Ohira T. (2017). Association between poor psychosocial conditions and diabetic nephropathy in Japanese type 2 diabetes patients: A cross-sectional study. J. Diabetes Investig..

[26] Karami H., Shiri M.S., Rezapour A., Mehrabadi R.S., Afshari S. (2021). The association between diabetic complications and health-related quality of life in patients with type 2 diabetes: A cross-sectional study from Iran. Qual. Life Res..

[27] Wang W.-L., Liang S., Zhu F.-L., Liu J.-Q., Wang S.-Y., Chen X.-M., Cai G.-Y. (2019). The prevalence of depression and the association between depression and kidney function and health-related quality of life in elderly patients with chronic kidney disease: A multicenter cross-sectional study. Clin. Interv. Aging.

[28] Pu L., Zou Y., Wu S.-K., Wang F., Zhang Y., Li G.-S., Wang J.-W., Zhang L.-X., Zhao M.-H., Wang L. (2019). Prevalence and associated factors of depressive symptoms among chronic kidney disease patients in China: Results from the Chinese Cohort Study of Chronic Kidney Disease (C-STRIDE). J. Psychosom. Res..

[29] Gleeson M., Bishop N.C., Stensel D.J., Lindley M.R., Mastana S.S., Nimmo M.A. (2011). The anti-inflammatory effects of exercise: Mechanisms and implications for the prevention and treatment of disease. Nat. Rev. Immunol..

[30] Khoo K., Man R.E.K., Rees G., Gupta P., Lamoureux E.L., Fenwick E.K. (2019). The relationship between diabetic retinopathy and psychosocial functioning: A systematic review. Qual. Life Res..

[31] Cukor D., Cohen S.D., Peterson R.A., Kimmel P.L. (2007). Psychosocial Aspects of Chronic Disease: ESRD as a Paradigmatic Illness. J. Am. Soc. Nephrol..

[32] Wium-Andersen M.K., Wium-Andersen I.K., Prescott E.I.B., Overvad K., Jorgensen M.B., Osler M. (2019). An attempt to explain the bidirectional association between ischaemic heart disease, stroke and depression: A cohort and meta-analytic approach. Br. J. Psychiatry.

[33] de Alencar S.B., de Lima F.M., Dias L.A., Dias V.A., Lessa A.C., Bezerra J.M., Apolinário J.F., de Petribu K.C. (2020). Depression and quality of life in older adults on hemodialysis. Braz. J. Psychiatry.

[34] Lever-van Milligen B.A., Vogelzangs N., Smit J.H., Penninx B.W. (2014). Hemoglobin levels in persons with depressive and/or anxiety disorders. J. Psychosom. Res..

[35] Al Naamani Z., Gormley K., Noble H., Santin O., Al Maqbali M. (2021). Fatigue, anxiety, depression and sleep quality in patients undergoing haemodialysis. BMC Nephrol..

[36] Campbell K.H., Huang E.S., Dale W., Parker M.M., John P.M., Young B.A., Moffet H.H., Laiteerapong N., Karter A.J. (2013). Association between Estimated GFR, Health-Related Quality of Life, and Depression Among Older Adults with Diabetes: The Diabetes and Aging Study. Am. J. Kidney Dis..

[37] Chiang H.-H., Guo H.-R., Livneh H., Lu M.-C., Yen M.-L., Tsai T.-Y. (2015). Increased risk of progression to dialysis or death in CKD patients with depressive symptoms: A prospective 3-year follow-up cohort study. J. Psychosom. Res..

[38] Zhang Z., He P., Liu M., Zhou C., Liu C., Li H., Zhang Y., Li Q., Ye Z., Wu Q. (2021). Association of Depressive Symptoms with Rapid Kidney Function Decline in Adults with Normal Kidney Function. Clin. J. Am. Soc. Nephrol..

[39] Tafet G.E., Nemeroff C.B. (2020). Pharmacological Treatment of Anxiety Disorders: The Role of the HPA Axis. Front. Psychiatry.

[40] Hedayati S.S., Finkelstein F.O. (2009). Epidemiology, Diagnosis, and Management of Depression in Patients with CKD. Am. J. Kidney Dis..

[41] Janssen M.F., Lubetkin E.I., Sekhobo J.P., Pickard A.S. (2011). The use of the EQ-5D preference-based health status measure in adults with Type 2 diabetes mellitus. Diabet. Med..

[42] Lee Y.-J., Kim M.S., Cho S., Kim S.R. (2013). Association of depression and anxiety with reduced quality of life in patients with predialysis chronic kidney disease. Int. J. Clin. Pract..

